# Myeloid Cells in Intact Human Cervical Explants Capture HIV and Can Transmit It to CD4 T Cells

**DOI:** 10.3389/fimmu.2018.02719

**Published:** 2018-11-23

**Authors:** Radiana T. Trifonova, Brooke Bollman, Natasha S. Barteneva, Judy Lieberman

**Affiliations:** Program in Cellular and Molecular Medicine, Department of Pediatrics, Harvard Medical School, Boston Children's Hospital, Boston, MA, United States

**Keywords:** cervix, myeloid cells, dendritic cells, HIV, transmission, cell sorting, imaging flow cytometry

## Abstract

The importance of myeloid cells in HIV transmission in the female genital tract is uncertain. Because it is difficult to study the early events in HIV transmission in humans, most of our knowledge is based on animal models of SIV infection in Rhesus macaques and more recently HIV infection in humanized mice. However, these models may not accurately recapitulate transmission in the human genital tract. CD14+ myeloid cells are the most abundant hematopoietic cells in the human cervical mucosa, comprising 40–50% of CD45+ mononuclear cells. Most CD14+ cells are CD14+CD11c– macrophages and about a third are CD14+CD11c+ tissue dendritic cells, which express the HIV-binding receptors, DC-SIGN and CX3CR1. To examine the role of mucosal myeloid cells in HIV transmission, we infected intact healthy human cervical explants with CCR5–tropic HIV-1 *ex vivo* and then sorted populations of cervical immune cells 20 h later to determine whether they took up virus and could transmit it to activated CD4 T cells. Viral RNA was detected in CD14+ myeloid cells in all but one of 10 donor tissue samples, even when HIV RNA was not detected in CD4+ T cells. HIV RNA was detected predominantly in CD14+CD11c+ dendritic cells rather than in CD14+CD11c– macrophages. The reverse transcriptase inhibitor, nevirapine, reduced HIV RNA in CD4+ T cells, but not in CD14+ cells. Moreover, integrated HIV DNA were not detected above background in myeloid cells but was detected in T cells. These data suggest that although HIV replicates in T cells, myeloid cells in the female genital mucosa capture viral particles, but do not replicate the virus at early timepoints. However, sorted CD14+ myeloid cells isolated 20 h post-infection from 5 HIV-infected cervical explants tested all transmitted HIV to activated CD4+ T cells, while only 1 sample of sorted CD4+ T cells did. Thus, myeloid cells in human cervical tissue capture HIV and are an important early cellular storage site of infectious virus.

## Introduction

Because studying sexual transmission of HIV in humans is difficult, models of heterosexual transmission are largely based on studies of SIV transmission in female Rhesus macaques (1, 2). In macaques, SIV first infects CD4+ T cells where it replicates before spreading to other cells in the tissue (1). However, results in the macaque SIV model might not translate to human HIV transmission. Immune genes have co-evolved with pathogens; in particular, innate immune genes in non-human primates and humans show signs of divergent evolution. Moreover, human and non-human primate microbiota in the female genital mucosa, which affect transmission (3), are different. Importantly SIV infection of primate immune cells differs from HIV-1. Although the efficiency of reverse transcription is reduced in non-dividing cells by the host factor SAMHD1, the Vpx accessory protein in SIV and HIV-2 overcomes SAMHD1's activity by promoting SAMHD1 degradation (4–7). Even amongst HIV strains and human subjects, HIV strain variations, vaginal infection and inflammation, and genetic variations strongly influence sexual transmission.

The immune cell composition of the human female genital mucosa influences susceptibility to HIV-1 transmission. In a previous study, we optimized a procedure that combined enzyme digestion and mechanical dissociation to retrieve both lymphoid and myeloid cells efficiently from the genital mucosa without removing cell subtype surface markers (8). A surprising finding was that CD14+ myeloid cells were the most abundant hematopoietic cells in the cervix, comprising about half of CD45+ mononuclear cells. Myeloid cells were located exclusively in the subepithelial stroma. Most of the CD14+ cells were conventional CD14+CD11c- macrophages, but about a third were CD14+CD11c+ tissue dendritic cells (DCs), most of which were CD103-CD11b+CX3CR1+DC-SIGN+. Previous studies had reported that myeloid cells represented only ~10% of the hematopoietic cells in the genital mucosa (9, 10), but this apparent discrepancy could have been due to suboptimal recovery from the tissue stroma with routine protocols for tissue dissociation. Because of their abundance in the genital tract, myeloid cells and specifically DCs, expressing the HIV-binding co-receptors, CX3CR1 (the fractalkine receptor) and DC-SIGN, might be important in HIV transmission.

It is unclear which human cells first bind HIV and whether myeloid cells contribute to transmission. Some *in vitro* studies suggest that myeloid cells can capture HIV during mucosal transmission and can transfer the virus to T cells and enhance dissemination to lymphoid tissue (11, 12). Thus, although myeloid cells do not efficiently replicate HIV-1 [reviewed in (13)] they could still be one of the first cells to take up the virus. Early myeloid cell viral capture could play an important role in transmission both by sensing the virus and inducing innate and adaptive immune responses and by transferring the virus to T cells (14, 15). Experiments using human intestinal explant models have suggested a role of myeloid cells in HIV transmission at intestinal epithelia (16, 17). In one study lamina propria DCs in human intestinal explants transported HIV-1 inoculated onto the apical surface through the mucosa and transmitted it in trans to blood and intestinal lymphocytes (16). Another study showed that lamina propria DCs, but not macrophages, in the gut can migrate toward R5-tropic virus to sample luminal virions, retain the virus and thereafter transmit the infection to receptive target cells (17). Furthermore, a study using single cell suspensions of cells from the lower female genital tract showed that DCs were the first cells to capture the virus, but HIV became predominant in T cells at later time points (18). Another study from the same group demonstrated that vaginal DCs capture transmitted founder HIV and that vaginal DCs, but not macrophages or CD3+ T cells, transport HIV out of the mucosa and could transfer HIV to vaginal and blood T cells (19). The same group also showed more recently that CD14+CD11c+ DCs derived from the human genital tract are one of the first immune cells to encounter HIV when a cell suspension of digested tissue is incubated with GFP-labeled HIV-viral-like particles (20) and that ovarian CD14+ cells could be infected with HIV (21).

To examine the cells that first capture HIV within intact female genital tissue, an important site of HIV heterosexual transmission, in this study we looked at infection in explants of human cervical mucosa that preserve the local tissue environment. We infected healthy donor human cervical tissue explants with JRCSF, a CCR5-tropic clinical isolate of HIV-1, to ask which cells are initially infected. In particular we wanted to know whether HIV-1, like SIV, first infects CD4+ T cells and amplifies in them. In some experiments, we compared infection of JRCSF packaged with Vpx (Vpx-JRCSF) with wild-type (WT) JRCSF to examine the role in mucosal infection of the HIV restriction factor SAMHD1, whose degradation is orchestrated by Vpx (6, 7). To capture the first infected cells, we sorted subpopulations of genital immune cells 20 h after infection and used sensitive qRT-PCR to look at which cell populations contain HIV RNA. HIV RNA was detected in CD14+ myeloid cells more often than in CD4+ T cells, suggesting that myeloid cells take up HIV early in transmission. Higher levels of HIV RNA were measured in samples infected with Vpx-JRCSF than with wild-type virus. HIV RNA was present predominantly in CD14+CD11c+ dendritic cells, rather than in CD11c-CD14+ macrophages. Imaging flow cytometry (IFC) confirmed preferential HIV uptake in cervical myeloid cells 1 day after infection. Myeloid cells took up the virus, but did not produce new viral particles, because viral RNA levels in CD14+ cells were not reduced by an RT inhibitor, as they were in CD4+ T cells. We also could not detect any signal for integrated provirus in myeloid cells by nested Alu-*gag* PCR, further suggesting that captured virus in myeloid cells did not replicate at this early timepoint. However, CD14+ myeloid cells from HIV-infected cervical explants transmitted the virus to activated blood CD4+ T cells, confirming that the virus taken up by the myeloid cells is infectious. Thus, resident tissue myeloid cells in the human genital tract mucosa may be the first cells to take up HIV and likely play a role in mucosal transmission.

## Materials and methods

### Human cervical tissue explants

Human cervical tissue was obtained from women without cervical pathology undergoing hysterectomy for benign conditions, such as fibroids, as previously described (8). The tissue was stored at 4°C in RPMI 1640 media (Cellgro) containing 10% Human Serum (AB) (GemCell), 100 U/ml penicillin and streptomycin sulfate 100 μg/ml (H10 medium) until processing within 24 h of surgery.

### HIV infection of cervical explants and peripheral blood CD4T cells

The cervical mucosa, separated from the underlying stroma by cutting 5 mm below the epithelial surface, was minced into 5 mm^3^ pieces and 10–15 pieces/well were placed in H10 medium in 12-well tissue culture plates (Falcon, BD). Samples were infected with R5-tropic HIV strain JRCSF or JRCSF virus packaged with Vpx. HIV-1 stocks were produced by the Virology Core of the Ragon Institute as previously described (22). JRCSF virus packaged with Vpx was produced using pIRES-GFP-Vpx provided by Dr. Mario Stevenson (4). The cervical explants were infected with 1.5 × 10^5^ TCID50/mL virus in 0.5 mL H10/well. Blood CD4+ T cells, pre-activated with 5 μg/mL Phytohaemagglutinin (PHA) (Difco) for 1 d at 1 × 10^6^ / mL and infected with 1.5 × 10^5^ TCID50/mL JRCSF in H10 containing 120 IU/mL recombinant human IL-2 (Proleukin, Chiron Corporation), served as a positive control. In experiments where HIV DNA was measured in sorted cells, the viral stock was pretreated with 2 IU/mL TURBO DNase (ThermoFisher Scientific) for 30 min at 37°C to remove residual plasmid from the viral preparation. In some experiments, 25 μM Non-Nucleoside RT Inhibitor nevirapine (NIH AIDS Research and Reference Reagent Program) or an equivalent volume of DMSO as control was added to the cervical explants 60 min before HIV infection.

### Cervical tissue digestion

A single cell suspension was prepared from cervical explants as described (8, 23). Briefly, 5 mm^3^ cervical tissue pieces were placed in pre-warmed 5 mg/ml Collagenase IV (Life Technologies) in H10 medium in a gentleMACS C Tube and subjected to mechanical dissociation for 1 min using mouse spleen 01.01 program of the gentleMACS Dissociator (MACS Miltenyi Biotec). To collect any cells that might have migrated out of the tissue, we centrifuged the culture media and included any pelleted cells in the cell suspension. The sample was then incubated for 30 min at 37°C with shaking at 150 rpm, followed by another cycle of mechanical dissociation. The suspension was filtered through a 100 μm cell strainer (BD Biosciences) and viable cells were collected by centrifugation at 500 × g for 5 min at 4°C.

### Cell sorting

Immune cells were identified by staining with antibodies for hematopoietic cells (anti-CD45), T cells (anti-CD3, anti-CD4 and anti-CD8), and myeloid cells (anti-CD14, anti-CD11c) with the following antibody cocktail: anti-CD45-Pacific blue (cl.HI30), anti-CD3-PerCP-Cy5.5 (cl.UCHT1), anti-CD8-PE (cl.HIT8a), anti-CD4-AF488 (cl.OKT4), anti-CD14-AF647 (cl.HCD14), and anti-CD11c-PE-Cy7 (cl.3.9) All antibodies were from BioLegend. In experiments where we used Vpx-JRCSF we did not include anti-CD4-AF488 because the virus expressed GFP-Vpx. (Although the signal for GFP-Vpx was too weak to detect, we excluded AF488 antibody from the staining panel to avoid possible interference with CD4 staining). We always included CD8 staining to differentiate CD8 T cells and CD4 T cells (CD8-negative CD3+) because HIV infected T cells down-regulate CD4 and might appear CD4–. Staining was performed in FACS buffer (Dulbecco's Phosphate-Buffered Saline, 1 mM EDTA, 25 mM HEPES (all from Invitrogen) with 0.5% BSA (Sigma) following incubation with an FcR block (TruStain, BioLegend) for 10 min at 4°C in FACS Buffer. Cells were incubated with antibodies for 30 min at 4°C, then washed with 2 ml of FACS buffer and centrifuged to pellet the cells. Dead cells were excluded using Sytox blue dead stain dye (Invitrogen). Live CD45+ cells stained with Pacific blue were well separated from the extremely bright Sytox blue+ dead cells that fluoresce in the same channel. T cells and myeloid cell subsets were sorted using a biocontained FACSARIA II. Cell sorting was performed in RPMI-1640 medium with L-Glutamine and without phenol red (Sigma-Aldrich) to preserve cell viability during sorting. The medium was reconstituted on the day of sorting in deionized water and supplemented with 25 mM HEPES (Gibco), 1 mM EDTA (Invitrogen), and filter sterilized using 0.45 μ filter bottles (ThermoFisher). Data were analyzed using FlowJo version 9.3.1 software (Tree Star).

### Quantitative RT-PCR

Total RNA was isolated from sorted cells using TRIZOL reagent and cDNA was synthesized using Superscript III and random hexamers (Invitrogen) following DNase (Roche) treatment (1 U per μg RNA) for 30 min at 37°C. Quantitative Real Time PCR was performed using SsoFast EvaGreen Supermix and a Bio-Rad CFX96 Real-Time PCR System (Bio-Rad Laboratories). The primers were as follows: *GAPDH* forward: 5′-AGCCACATCGCTCAGACAC-3′, *GAPDH* reverse: 5′-GCCCAATACGACCAAATCC-3′, *CD4* forward: 5′-GGCAGTGTCTGCTGAGTGAC-3′, *CD4* reverse: 5′-GACCATGTGGGCAGAACCT-3′, *CD3* forward: 5′-CCTCTTATCAGTTGGCGTTTGG-3′, *CD3* reverse: 5′-TTCAGTGACAGGTGATCCTCA-3′. The primers for HIV *gag* were as described (24): GagABC forward: 5′-CCTAGGAAAAAGGGCTGTTGGA-3′, GagABC reverse: 5′-AGGAAGGCCAGATCTTCCCTAAA-3′. A control without Reverse Transcriptase was included in each experiment and was required to be negative to rule out plasmid DNA contamination. mRNA expression was normalized to *GAPDH* expression and then calculated as 2^−Δ*Ct*^, since no Ct value was available for *gag* for the uninfected control (25). An arbitrary value of 0.00001 was assigned to samples where *gag* was not above the limit of detection.

### Quantitative PCR

To detect viral DNA, total DNA was isolated using the DNeasy Kit (Qiagen) or Phenol-Chloroform Isoamyl Alcohol (Sigma-Aldrich) (26). Strong Stop (SS), late transcripts (gag) and 2-LTR circles were detected using the following primers: HIV strong stop (SS) forward: 5′-TCTGGCTAACTAGGGAACCCA-3′, HIV strong stop (SS) reverse: 5′-CTGACTAAAAGGGTCTGAGG-3′, HIV *gag* forward: 5′-ATGGTTGTAGCTGTCCCAGTATTTG-3′, HIV *gag* reverse:5′-ATAGTATGGGCAAGCAGGGAGCTA-3′, HIV 2LTR forward: 5′-GCCTGGGAGCTCTCTGGCTAA-3′, HIV 2LTR reverse:5′-GCCTTGTGTGTGGTAGATCCA-3′. PCR was performed in triplicate for each sample using SsoFast EvaGreen Supermix as described above. PCR conditions were as follows: initial denaturation at 95°C for 10 min followed by 40 rounds of cycling at 95°C for 15 s and 60°C for 60 s. To measure integrated HIV DNA, genomic DNA was isolated using *Quick*-gDNA^TM^ Microprep (Zymo Research Corp.). The following primers and probe were used for the nested Alu-gag PCR (27). For Round 1 PCR: *Alu* forward: 5′-GCC TCC CAA AGT GCT GGG ATT ACA G-3′, HIV *gag* reverse: 5′-GTT CCT GCT ATG TCA CTT CC-3′. For Round 2 qPCR: RU5 R forward: 5′-TTA AGC CTC AAT AAA GCT TGC C-3′, RU5 U5 reverse: 5′-GTT CGG GCG CCA CTG CTA GA-3′ and RU5 wildtype Probe: 5′-CCA GAG TCA CAC AAC AGA CGG GCA CA-3′. Data was presented as 2^−Δ*Ct*^ relative to the signal for *GAPDH*. The nested Alu-HIV PCR value was normalized to the unnested GAPDH DNA PCR value and hence does not represent a measurement of the absolute number of copies of integrated HIV DNA per cell. Because of this, the values for integrated provirus obtained by nested Alu-HIV PCR cannot be compared to the measurements for unintegrated HIV gag DNA species, but the values of integrated HIV in CD4+ T cells and CD14+ cells can be compared.

### Imaging flow cytometry

A single cell suspension was prepared from human cervical explants as described above 24 h post-infection with HIV-JRCSF. Cells were stained with anti-CD14-AF647 (cl.HCD14) (BioLegend) and then fixed and permeabilized prior to intracellular staining with phycoerythrin (PE)-conjugated mouse anti-p24 mAb (KC57-RD1, Beckman Coulter). Intracellular staining was performed using BD Cytofix/Cytoperm kit (BD Biosciences) with a modified fixation step (28). Fixation with 4% PFA preheated to 80°C significantly reduced the background for analysis of myeloid cells from cervical tissue samples and enabled us to separate positive signal from background. Therefore, samples were fixed with 4% PFA preheated to 80°C for 2 min before intracellular staining, which was performed using the Perm/Wash buffer provided with the BD Cytofix/Cytoperm kit following the manufacturer's recommendations. Nuclei were stained with 1 μg/ml DAPI (Sigma) for 10 min. Samples were acquired using a 5-laser ImageStream X Mark II imaging cytometer (Amnis-Millipore). A minimum of 20,000 events was recorded per sample. Image analysis was performed using Imagestream Data Exploration and Analysis Software (IDEAS) 6.1 (Amnis-Millipore). Cell populations were sequentially gated on single DAPI+ CD14+ or CD14- cells. The percent of p24 Ag+ cells in the infected samples were compared to uninfected control samples from the same donor to establish the level of background fluorescence.

### Co-culture of sorted human cervical cells and blood CD4+ T cells

Human PBMCs were isolated by Ficoll-Paque (GE Healthcare) density gradient centrifugation from whole blood. CD4+ T cells were separated from CD14+ PBMCs using CD14 (negative selection of lymphocytes) and CD4 (positive selection of CD4+ T cells) magnetic microbeads (MACS Miltenyi Biotec). Cells were cultured in H10 medium and activated with 5 μg/mL PHA for 3 d prior to co-culture with sorted cervical explant cells. The coculture protocol was adapted from Hollinger et al. (29). CD4+ T cells, CD8+ T cells and myeloid CD14+ cells were sorted from human cervical explants 20 h post-infection with JRCSF HIV and immediately used for co-culture with pre-activated blood CD4+ T cells. The average numbers of sorted cells were 2.25 × 10^4^ CD4+ T cells, 1.89 × 10^4^ CD8+ T cells, and 2.27 × 10^4^ CD14+ myeloid cells. Pre-activated CD4+ T cells (3 × 10^5^) were plated per well in U-bottom 96 well plates (Falcon, BD) and mixed with cells sorted from human cervical explants in 250 μl per well of H10 containing 120 IU/mL recombinant human IL-2. Plates were centrifuged for 2 min at 500 × g to bring the blood CD4+ cells and the cervical cells into contact and then cultured for 14 d. Blood CD4+ T cells alone infected with JRCSF HIV served as a positive control and uninfected cells served as a negative control. Every third day, the cells were centrifuged, and half of the media was collected and stored at −80°C prior to analysis by p24 Ag ELISA. An equal volume of fresh media containing 240 IU/mL IL-2 was added to each well. On day 7 of culture, 3 × 10^5^ fresh PHA pre-activated CD4+ T cells were added to each well.

### p24 antigen ELISA

p24 antigen in culture media was measured using the HIV-1 p24 ELISA Kit (Perkin Elmer). Absorbance was measured with a Synergy2 Biotek Microplate Reader (Winooski) using Gen5™ Reader Control and Data Analysis Software. The lower Limit of Detection of the assay was calculated as Mean + 2^*^Standard Deviation of the negative control (culture medium alone).

### Statistical analysis

Paired two-tailed *t*-test, was used to compare infection of different cell populations in explants infected with HIV strain JRCSF and to compare infection with WT JRCSF and JRCSF packaged with Vpx. All statistical analysis was performed using GraphPad Prism version 6.00 for Mac OS X (GraphPad Software, La Jolla California).

### Ethics statement

Human cervical tissue was obtained with Institutional Review Board approval as previously described (8). The study did not require consent because the tissue samples used were discarded post-surgery and collected anonymously. Cervical tissue samples were provided by the Tissue and Tumor Bank at University of Massachusetts Medical School (Worcester) and by the Cooperative Human Tissue Network (CHTN), National Cancer Institute. Anonymized whole blood samples were obtained from the Brigham and Women's Hospital Specimen Bank, Boston, MA with Institutional Review Board approval.

## Results

### HIV RNA is detected in human cervical myeloid cells 20 h after infection

**Figure 1:**
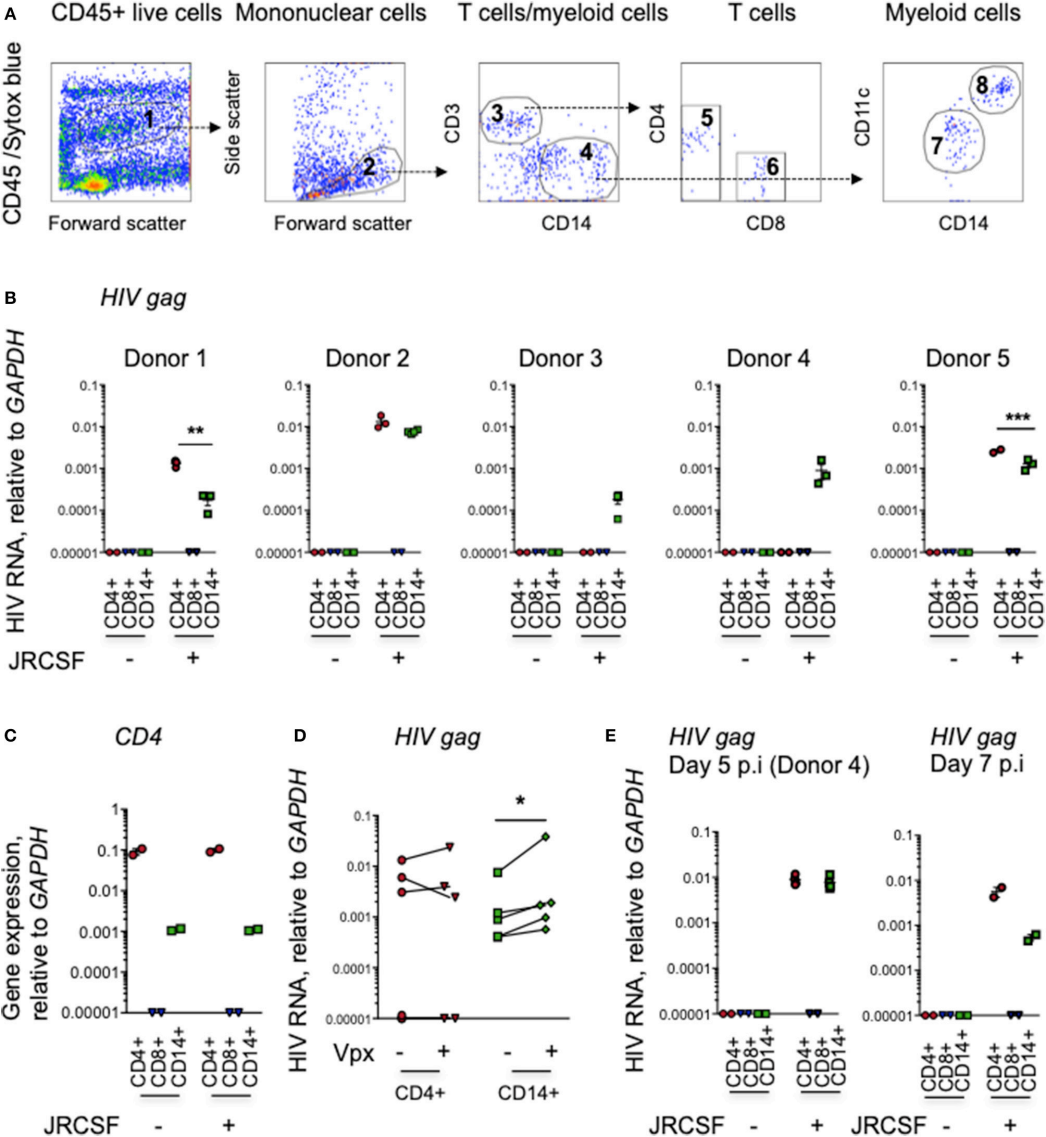
Myeloid cells in human cerival explants take up HIV. **(A)** Scheme for sorting immune cells from human cervical tissue. The figure shows 10,000 events recorded before cell sorting to set gates in a representative single cell suspension from a human cervical explant. The CD45+ cell gate (1) excluded CD45- and Sytox blue-stained dead cells. The mononuclear cell gate (2) based on forward and side scatter. CD3+ T cells (3) and CD14+ myeloid cells (4). CD3+ T cells from gate 3 separated into CD4+ (5) and CD8+ (6) T cells. CD14+ myeloid cells from gate 4 separated into CD11c– (7) macrophages and CD11c+ dendritic cells (8). **(B)** HIV RNA in cervical immune cells. HIV infection, assessed by qRT-PCR for *gag*, relative to *GAPDH*, in CD3+ CD4+ and CD8+ T cells and CD14+ myeloid cells, sorted from human cervical explants from five donors 20 h post-infection with HIV strain JRCSF and in uninfected control tissues. The lines indicate mean and SEM. **(C)** CD4 expression, measured by qRT-PCR, in sorted cervical CD4+ T cells, CD14+ myeloid cells, and CD8 T cells (representative data from donor 3). **(D)** Comparison of HIV RNA levels, assayed by *gag* qRT-PCR relative to *GAPDH*, in sorted cervical CD3+CD4+ T cells and CD14+ myeloid cells sorted from five donors 20 h post-infection with JRCSF or JRCSF packaged with Vpx protein. **(E)** HIV *gag* RNA by qRT-PCR in CD3+CD4+ T cells, CD14+ myeloid cells and CD3+CD8+ T cells sorted from uninfected or infected human cervical explants from donor 4, 5 days post-HIV infection (p.i.) and two additional cervical explants tested 7 days p.i. qRT-PCR values below the level of detection were arbitrarily assigned the value 0.00001. ^*^p < 0.05, ^**^p < 0.01, ^***^p < 0.001, paired *t*-test.

To determine which human cells are initially infected with HIV, we infected intact human cervical explants from 5 healthy donors with CCR5-tropic JRCSF HIV-1 and 20 h later prepared single cell suspensions and sorted myeloid cells and T cells and analyzed their HIV *gag* RNA content by qRT-PCR. To test the effect of SAMHD1 degradation by Vpx on viral replication in mucosal myeloid and T cells, we also infected with Vpx-JRCSF. Our gating strategy for sorting CD4+ and CD8+ T cells and CD14+ myeloid cells is shown in Figure 1A. All CD8-CD3+ cells were included in the CD4 T cell gate, because HIV-infected cells down-regulate CD4 and can appear CD4^dim^ or CD4^−^. In all 5 donors, we readily detected HIV RNA in myeloid cells (Figure 1B). In 3 of 5 donors, we also detected HIV RNA in CD4 T cells, but not in CD8 T cells, as expected. Thus, HIV RNA was detected in CD14+ cells even when it was not detected in CD4+ T cells. The purity of the sorted populations was confirmed by measuring *CD4* mRNA by qRT-PCR; the sorted CD4+ T cell samples had much higher *CD4* expression than the CD14+ myeloid cells, and *CD4* mRNA was not detected in the sorted CD8+ T cells (Figure 1C). Significantly higher levels of HIV RNA were detected in myeloid cells in the cervix after infection with JRCSF-Vpx than with WT JRCSF (*p* = 0.02, paired *t*-test), but the amount of HIV RNA did not significantly differ in CD4+ T cells (*p* = 0.78) (Figure 1D). In one of the samples in which HIV RNA was not detected in CD4 T cells at 20 h (donor 4), we also examined infection 5 days after infection (Figure 1E). HIV was still detected in CD14+ cervical cells 5 days post-infection, but now was also detected in CD4 T cells. In two additional samples tested at 7 days post-infection HIV was detected in CD4 T cells and still detected in CD14+ cells (Figure 1E).

### HIV is captured mostly by CD14+CD11C+ tissue dendritic cells

**Figure 2:**
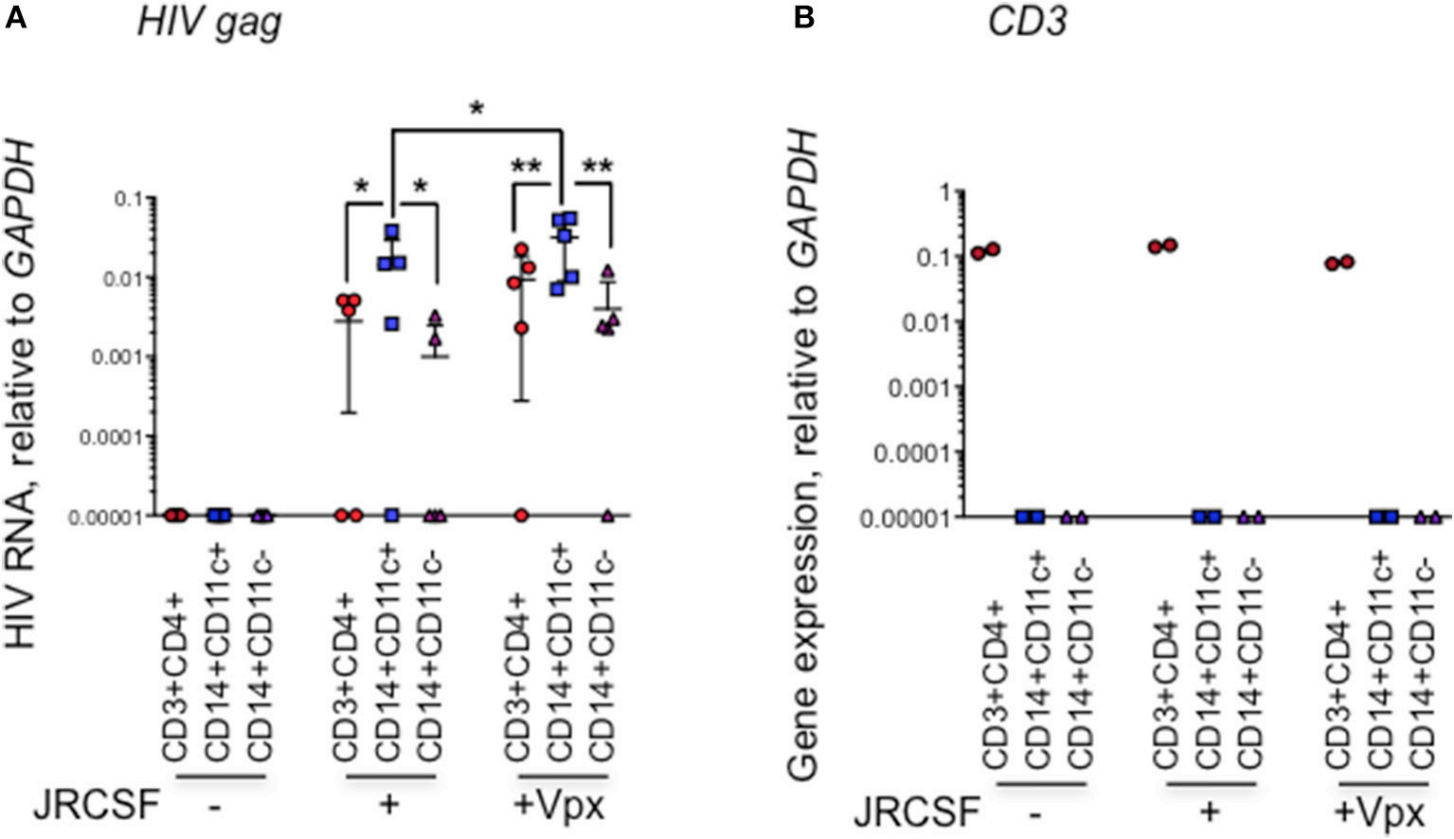
HIV RNA is more abundant in CD14+CD11c+ dendritic cells than CD14+CD11c– macrophages in infected human cervical explants. **(A)** HIV infection measured by *gag* qRT-PCR relative to *GAPDH* in sorted cells from human cervical explants 20 h post-infection with HIV strain JRCSF or JRCSF packaged with Vpx protein and uninfected control samples. Indicated are mean and SD for five healthy donor tissues (each dot represents data from an individual donor). **(B)** *CD3* expression, measured by qRT-PCR (representative data from one donor) was used to confirm the purity of the sorted populations. qRT-PCR values below the level of detection were arbitrarily assigned the value 0.00001. ^*^p < 0.05, ^**^p < 0.01, paired *t*-test.

We next looked at which myeloid cell subset in the human cervix takes up HIV. In 5 additional HIV-infected normal donor cervical explants, we used qRT-PCR to amplify HIV RNA from sorted cervical CD3+CD4+ T cells, CD14+CD11c+ DCs, and CD14+CD11c- macrophages (8) harvested 20 h post-infection. In the myeloid compartment, HIV RNA was detected predominantly in CD14+CD11c+ tissue DCs in the cervix (Figure 2A). HIV RNA was detected above background in the CD14+CD11c+ cells from 4 to 5 samples infected with WT-JRCSF HIV. Lower levels of HIV RNA were detected in CD3+CD4+ T cells from 3 to 5 donors and in CD14+CD11c- macrophages from 2 to 5 donors infected with WT-JRCSF. HIV RNA was significantly higher in the DCs in explants infected with Vpx-JRCSF compared to WT virus (*p* = 0.01, paired *t*-test). There was no significant difference in HIV RNA in CD4+ T cells or macrophages in the explants infected with Vpx-JRCSF compared to WT virus. To confirm that HIV RNA detection in CD14+ populations could not be explained by contamination with CD4 T cells, we measured *CD3* mRNA by qRT-PCR in the sorted cells and found that the sorted cells were not contaminated with CD3-expressing T cells (Figure 2B).

### Detection of HIV p24 Ag in human cervical myeloid cells by imaging flow cytometry

**Figure 3:**
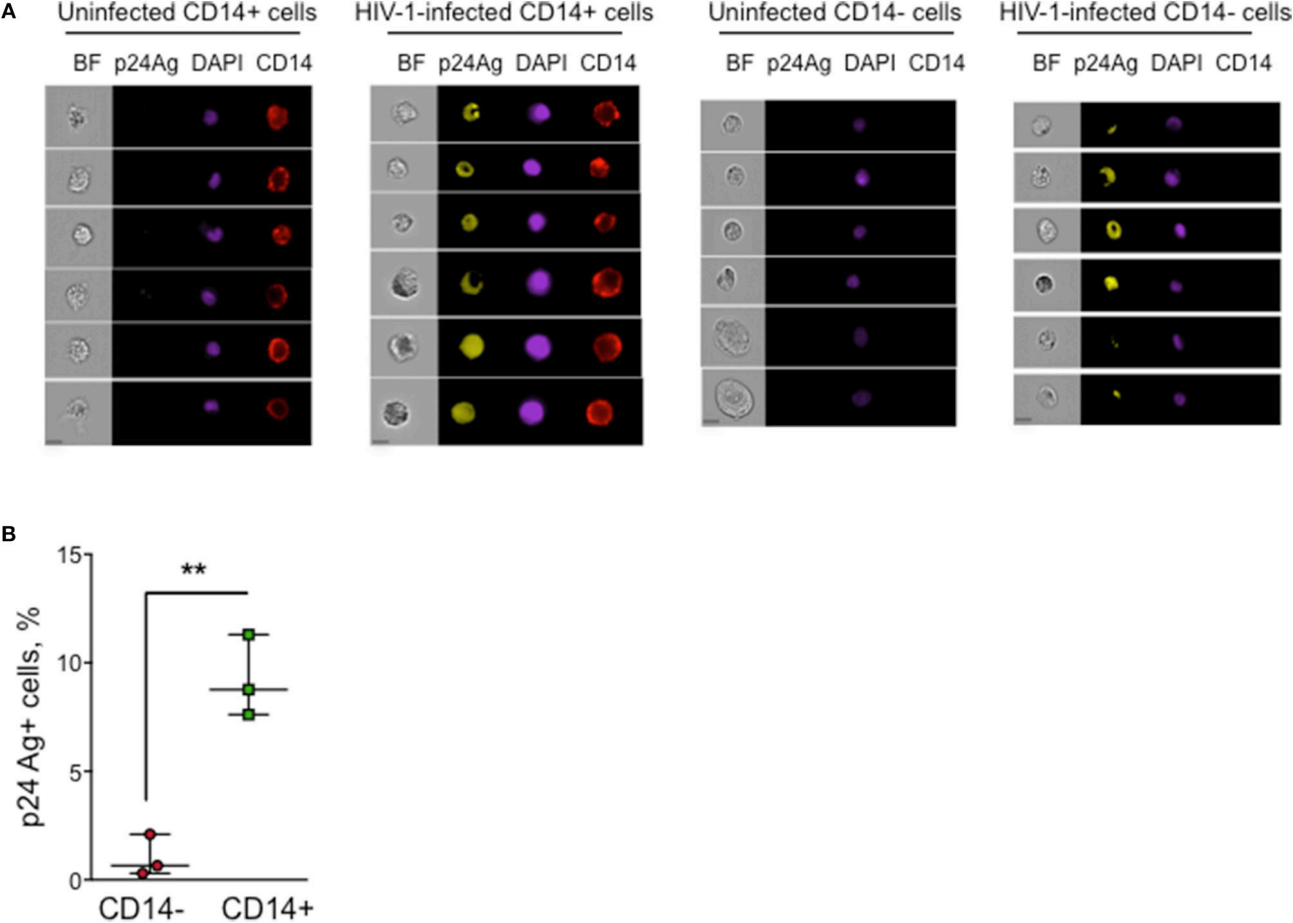
p24 Ag+ myeloid cells in human cervical explants analyzed by Imaging Flow Cytometry. Intracellular HIV- p24 Ag staining of a single cell suspension prepared from human cervical explants 20 h post-infection with HIV strain JRCSF. Myeloid cells were identified by cell surface CD14 staining. **(A)** Shown are representative images of uninfected and infected CD14+ and CD14- cells in one of three donor samples stained for CD14, p24 Ag, and DAPI (BF, brightfield image, scale bar 7 μm). **(B)** Percent of p24 Ag+ CD14– and CD14+ cells in HIV-infected cervical explants from three donors (each dot represents data from an individual donor). The lines indicate median and range. ^**^p < 0.01, paired *t*-test.

To determine the percent myeloid and lymphoid cells in the human cervix that captured HIV, we next used IFC to analyze single cell suspensions prepared from HIV-infected human cervical explants from an additional 3 donors harvested 24 h post-HIV infection, which were stained for cell surface CD14 and intracellular p24 Ag. We limited the number of fluorochromes used to reduce the risk of bleed-through fluorescence into the p24 Ag channel. Thus, we did not stain for any T cell markers, but used the absence of CD14 and cell size and morphology to identify T cells. Intracellular p24 Ag was detected above background in CD14+ cells in all 3 donors (Figures 3A,B). The percent of p24 Ag+ cells ranged between 7.6 and 11.3% in CD14+ myeloid cells and between 0.3 and 2.1% in CD14- T cells (*p* = 0.006, paired *t*-test). The low percent of HIV+ lymphoid vs. myeloid cells at this early time post-infection is consistent with our qRT-PCR data.

### HIV does not replicate in cervical myeloid cells

**Figure 4:**
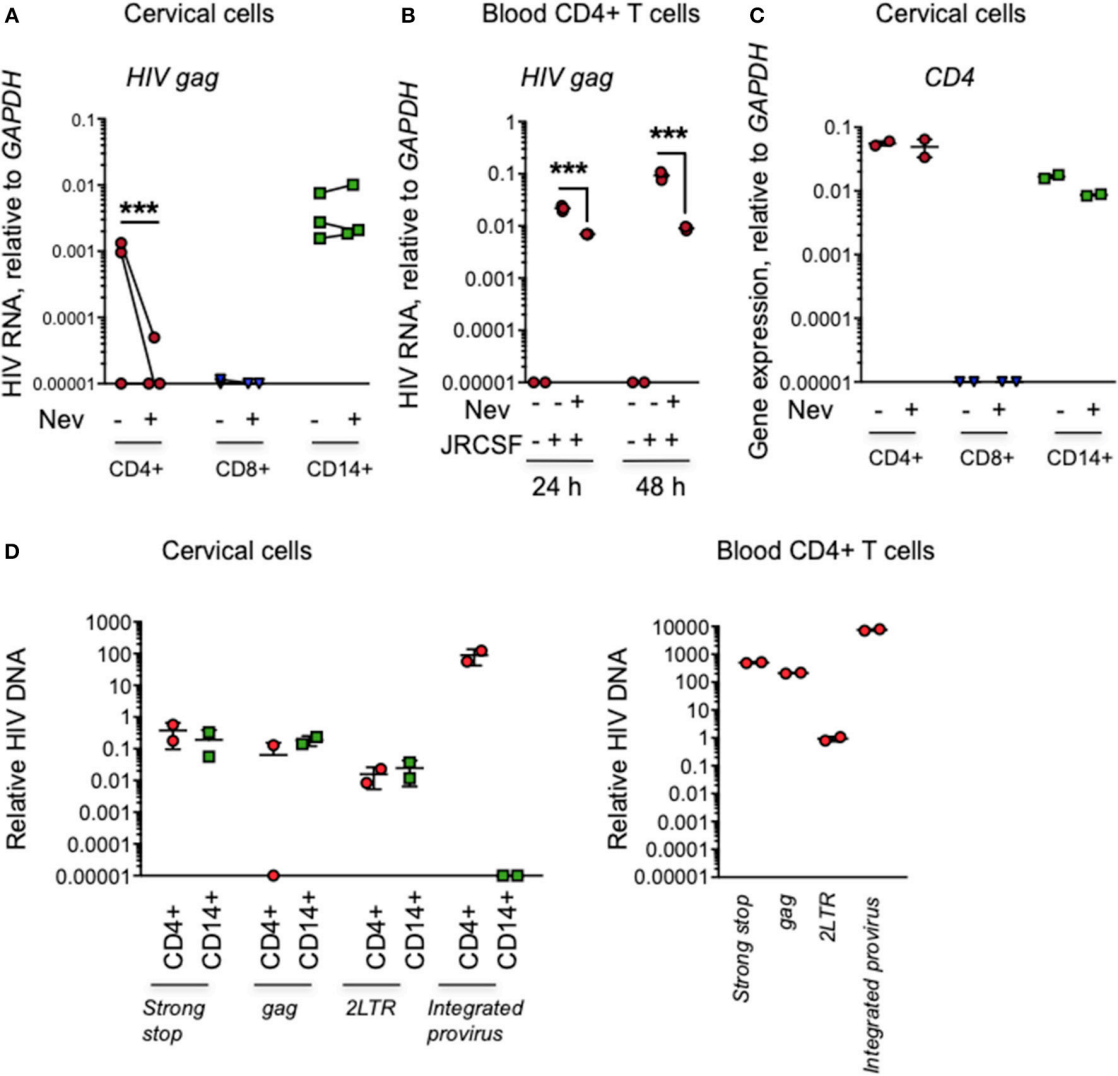
Myeloid cell HIV in infected cervical explants is not affected by nevirapine and does not efficiently integrate at 24 h post-infection. **(A)** HIV RNA assessed by qRT-PCR for HIV *gag*, relative to *GAPDH*, in CD3+CD4+ T cells, CD14+ myeloid cells and CD3+CD8+ T cells sorted from human cervical explants from three donors 24 h post-infection with HIV strain JRCSF. Tissues were pretreated with nevirapine (Nev) or DMSO control (each dot represents data from an individual donor). **(B)** HIV RNA measured by qRT-PCR for viral gag in human peripheral blood CD4+ T cells 24 and 48 h post-infection with HIV strain JRCSF treated with Nevirapine as a control for the RT inhibitor activity. **(C)** CD4 expression measured by qRT-PCR relative to *GAPDH* in CD4+ T cells (CD4 bright), CD14+ myeloid cells (CD4 dim), and CD8 T cells (CD4 negative) from uninfected human cervix to confirm the purity of the sorted populations (data are representative from 1 of 3 donors). **(D)** HIV DNA species (strong-stop DNA, late reverse transcription products, 2-LTR circles) measured by qPCR and HIV integrated provirus measured by nested Alu-PCR in CD14+ myeloid cells and CD3+CD4+ T cells sorted from human cervical explants (left) or activated peripheral blood CD4+ T cells (right) 24 h post-infection with HIV strain JRCSF (mean and SD of data from two donors). Data are normalized relative to qPCR for *GAPDH*. Because the normalization of integrated HIV DNA did not use a nested PCR control, the number of copies per cell of integrated HIV DNA in the different cell types is only relative and cannot be reliably compared to the number of copies of unintegrated HIV reverse transcripts. qRT-PCR values below the level of detection were arbitrarily assigned the value 0.00001. ^***^p < 0.001, paired *t*-test.

To determine whether HIV in cervical myeloid cells is produced or amplified by viral replication, we compared HIV infection by qRT-PCR 24 h post-infection in myeloid cells and CD4 and CD8 T cells in cervical explants from an additional 3 donors cultured in the presence or absence of the RT inhibitor nevirapine. In the absence of nevirapine, HIV *gag* RNA was detected in CD4 CD14+ myeloid cells from all donors and in CD4+ T cells from 2 of 3 donors, but not in CD8 T cells. Nevirapine did not significantly change HIV RNA levels in myeloid cells, but strongly and significant suppressed HIV RNA in CD4 T cells (Figure 4A). These data suggest that most of the viral RNA detected in T cells was produced by replicating virus, but the viral RNA detected in myeloid cells was input virus. As expected, nevirapine also strongly reduced HIV RNA in blood CD4 T cells (Figure 4B). The purity of the sorted populations was confirmed by qRT-PCR amplification of *CD4* (Figure 4C).

To examine more carefully whether HIV replication occurs in cervical myeloid cells, we also used qPCR to amplify viral DNA in sorted cells from 5 HIV-infected explants 24 h post-infection, using primers that detect early Strong Stop (SS) and late *gag* reverse transcripts and 2-LTR circles. Strong Stop (SS) and late transcripts (gag) were detectable in cervical myeloid cells, but 2-LTR circles were hardly detectable and integrated viral DNA assayed by nested Alu-gag PCR was not detected above background (Figure 4D, left). Activated human peripheral blood CD4+ T cells were infected with HIV for 24 h in parallel as a positive control for the qPCR assay (Figure 4D, right). Since reverse transcription is known to start within virions before cells are infected (30), it is hard to conclude much about viral replication in myeloid cells from these data. Viral SS and late *gag* reverse transcript levels in tissue CD4+ T cells were not statistically different from levels in tissue myeloid cells and 2-LTR circles were close to background. However, although we didn't detect integrated HIV DNA in myeloid cells 24 h post-infection, we detected integrated HIV DNA in CD4+ T cells, consistent with productive infection in CD4 T cells but not in myeloid cells at this early timepoint.

### Cervical myeloid cells transfer HIV to CD4 T cells

**Figure 5:**
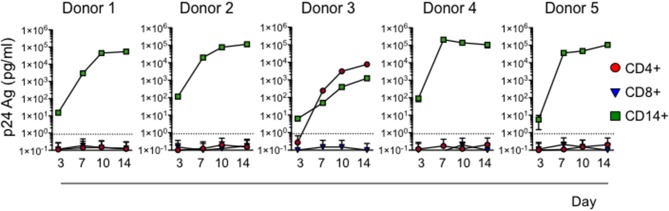
Cervical CD14+ cells reproducibly transmit HIV to activated CD4+ T cells. HIV infection was assessed by measuring p24 Ag in media at indicated times of activated human blood CD4+ T cell co-culture with sorted CD3+CD4+ T cells, CD3+CD8+ T cells, or CD14+ myeloid cells harvested from dissociated human cervical explants 20 h post-infection with HIV strain JRCSF. Dotted line represents the Limit of Detection of the assay (0.86 pg/mL).

To assess whether HIV captured by cervical myeloid cells is infectious, we co-cultured sorted myeloid cells, CD4 T cells and CD8 T cells from 5 HIV-infected normal human donor cervical explants, harvested 20 h post-infection, with heterologous HIV-susceptible activated blood CD4 T cells, using a sensitive assay developed to detect latent HIV infection in human blood cells (29). HIV infection was evaluated by measuring HIV p24 Ag in culture media over 14 days. In all five donors, co-culture with cervical CD14+ cells transferred HIV infection, but CD4 T cells from only one sample contained infectious virus (Figure 5). As expected, none of the sorted cervical CD8 T cells transmitted the infection. Thus, CD14+ myeloid cells sorted from infected cervical explants 1 day after infection are more efficient at transmitting infectious HIV to activated CD4+ T cells than cervical CD4 T cells, suggesting an important role of myeloid cell capture early in HIV transmission.

## Discussion

In this study, we infected intact human cervical explants with HIV to identify the first cells in the human cervix that capture HIV. Our data suggest that myeloid cells play a major role in the earliest events of HIV transmission. In the first day post-exposure to virus, tissue-resident CD14+CD11c+ DCs were the dominant cell type that captured HIV in the cervical mucosa. The virus captured in myeloid cells did not appear to integrate or replicate in myeloid cells since nevirapine did not reduce myeloid cell viral levels and integrated pro-virus was not detected in myeloid cells at this early timepoint. In contrast, these assays performed on CD4 T cells in the tissue in parallel detected evidence of reverse transcription and integration. However, captured HIV in myeloid cells could infect CD4 T cells in trans in all samples tested without being productively infected. Although integrated HIV DNA was not detected in the first day after infection, integrated pro-viral DNA could be amplified in myeloid cells harvested 5 days after infection (data not shown), suggesting either that viral integration is inefficient or slow in myeloid cells compared to T cells in freshly isolated tissue or that tissue myeloid cells only become capable of replicating the virus later as the infection spreads and the tissue becomes inflamed.

Our findings are consistent with a previous study, which used immunohistochemistry to detect HIV in human cervix 7 days post-infection and reported that HIV p24 Ag+ cells were predominantly CD14+/CD68+ cells and only a few were CD3+ T cells (31). They are also consistent with more recent studies that used intestinal tissue explants (16, 17) or genital tract tissue cell suspensions (18–21) to suggest a crucial role of mucosal DCs in the early capture of HIV. An advantage of our study is that we looked at infection of resident primary myeloid cells in the intact human cervix early after infection, unlike earlier studies which used blood derived *in vitro* differentiated macrophages and DCs or intestinal primary DCs, which have a different phenotype from genital tract cells (32). Our experimental model using cervical explants also better mimics the conditions of HIV mucosal tissue infection compared to infection of tissue cells in suspension. Enzymatic tissue digestion to produce single cell suspensions prior to infection can cleave cell surface markers that could affect HIV binding capacity (8). Since we digested the tissue after infection at the time of analysis, the virus was allowed to interact with intact tissue with preserved tissue architecture and cell surface receptors. Another advantage of our study is that we infected the explants with a clinical isolate of HIV (JRCSF) rather than lab strain viruses. We also used sensitive quantitative PCR to analyze different stages of HIV replication and to distinguish productive and non-productive infection in different cell subsets.

The levels of HIV RNA in myeloid cells did not change in the presence of nevirapine, suggesting that HIV did not replicate in these cells. In contrast, nevirapine inhibited HIV RNA detection in cervical CD4 T cells by more than a log, suggesting that most of the viral RNA detected in T cells was the result of productive infection. The CD4:CD8 T cell ratio was reduced on average by 30% 20 h post-infection and by 70% by Day 5 post-infection. The relative loss of CD4 T cells is unlikely to be secondary to their migration from the tissue, since we collected any migrated cells. Active viral infection in T cells in the explants could explain the loss of CD4 T cells in HIV-infected explants. However, our methods cannot determine how much T cell death could be attributed to “bystander” death of T cells that were either uninfected or in which viral replication was non-productive (33). Viral RNA levels remained high in myeloid cells at least for 7 days post-infection (Figure 1E) suggesting that dendritic cells might serve as a source of infectious virus at least in the first week of infection. Other myeloid cells, including alveolar macrophages, liver macrophages and CNS microglia, have been shown to serve as HIV reservoirs (34–38). A recent study in the humanized mouse model also confirmed that tissue macrophages are an HIV reservoir (39). Thus, cervical myeloid cells might also serve a role in establishing a viral reservoir. Since tissue DCs traffic to draining lymph nodes, DCs that take up virus might also play a role in viral systemic dissemination from the genital tract in humans. It would be worth examining this question in humanized mice. However, improved humanized mouse models that have better myeloid cell engraftment than first generation models of transplanted fetal bone marrow, liver, and thymus might be necessary to do this. This should be possible by transplanting these hematopoietic tissues into mice expressing human myeloid cell growth factors (40).

How myeloid cells take up HIV during mucosal infection is not completely certain. *In vitro* studies have demonstrated that HIV can be stored in invaginations of the plasma membrane in DCs that infect T cells in trans (41, 42). DC-SIGN and CX3CR1 lectin receptors expressed by myeloid cells are known to bind HIV (11, 43) and DC-SIGN has been shown to be important not only for HIV capture, but also for the transfer of HIV to T cells (44). DCs can also take up HIV by binding of the DC sialoadhesin CD169/Siglec-1 to a ganglioside on the viral membrane (45). A recent study showed that sinus-lining macrophages in humanized mice model capture HIV-1 through CD169/Siglec-1 (46). CD169 may be involved in HIV uptake by cervical DCs since they express the receptor (data not shown). Although macrophages can also be infected through engulfment of infected T cells (47), that is unlikely to be the mechanism of myeloid cell infection within the first day of exposure in our study, since myeloid cell infection sometimes occurred without T cell infection and was not affected by nevirapine.

When we evaluated the role of Vpx in human cervical myeloid cell infection, we consistently detected higher levels of HIV RNA in dendritic cells in explants infected with JRCSF packaged with Vpx protein than with wild type virus. This is not surprising since Vpx should allow for some viral replication in myeloid cells. However, Vpx, which should also promote HIV replication in resting T cells, did not appear to have a significant effect on viral levels in T cells, suggesting that SAMHD1 may not have been an important HIV restriction factor in tissue-resident T cells at least at an early time point after infection.

Our study suggests that CD14+ myeloid cells in the genital tract play an important role in heterosexual transmission of HIV in humans. Knowledge of the role of myeloid cells in the genital tract during HIV infection should inform the development of prevention strategies for HIV mucosal transmission and treatment strategies for reservoir elimination such as pharmacological inhibition of viral transfer from myeloid cells to T cells (48).

## Author contributions

JL and RT designed the research plan and wrote the manuscript. RT performed the experiments with cervical explants, cell sorting, qRT-PCR, and p24Ag ELISA. BB assisted with the PCR experiments. RT and NB designed, performed and analyzed the IFC experiments.

### Conflict of interest statement

The authors declare that the research was conducted in the absence of any commercial or financial relationships that could be construed as a potential conflict of interest.
